# Facilitators and Barriers to Engaging Stakeholders in Use-Inspired Aging Research

**DOI:** 10.1093/geroni/igaa057.728

**Published:** 2020-12-16

**Authors:** Rose Ann DiMaria-Ghalili, Justine Sefcik, Katherine Clark, Kathleen Fisher, Jina Huh-Yoo, Jana Hirsch, Laura Gitlin

**Affiliations:** 1 Drexel University, Jenkintown, Pennsylvania, United States; 2 Drexel University, Philadelphia, Pennsylvania, United States; 3 Drexel University College of Nursing and Health Professions, Philadelphia, Pennsylvania, United States; 4 Drexel University School of Public Health, Philadelphia, Pennsylvania, United States

## Abstract

The Cell2Society Aging Research Network at Drexel University is a university-wide, novel ecosystem for the pursuit of use-inspired aging research focused on topics from cellular to societal level. Our goals include: 1) Engaging stakeholders to jointly participate in age-related research; 2) Collaborating to build an infrastructure for age-related research through team science; 3) Develop and implement person-centered research that matters to older adults. We apply interdisciplinary, use-inspired approaches in three areas of relevance to older individuals, their families, healthcare and payment systems, communities and policy makers: (a) preventing and managing chronic conditions, (b) enhancing active and purposeful living, and (c) enabling aging in place in home and communities. Our initial qualitative study described facilitators and barriers experienced by community-based service providers (N=9) and payers/providers (N=5) in the greater Philadelphia area when engaging with academic investigators. We conducted three focus groups. Participants were mostly female (64%), white (64%) and were at their organization between 1-10 years (79%).Conventional content analysis revealed that successful research partnerships were facilitated by: 1) trusted investigators and academic institutions and 2) demonstrated collaborative qualities of investigators (e.g., good communication; provides staff education). Negative experiences engaging with academic-led research were related to: 1) research leaving the community (e.g., results never being shared) and 2) organizational limitations (e.g., lack of internal resources to complete projects). These findings will inform the development of stakeholder-academic partnerships from 2020 onward to design use-inspired aging research initiatives.

